# Mexican Plants and Derivates Compounds as Alternative for Inflammatory and Neuropathic Pain Treatment—A Review

**DOI:** 10.3390/plants10050865

**Published:** 2021-04-25

**Authors:** Geovanna N. Quiñonez-Bastidas, Andrés Navarrete

**Affiliations:** Departamento de Farmacia, Facultad de Química, Universidad Nacional Autónoma de México, Ciudad Universitaria, Coyoacán, Ciudad de México 04510, Mexico

**Keywords:** neuropathic pain, inflammatory pain, chronic pain, natural compounds, Mexican plants, pain treatment

## Abstract

Despite the availability of many anti-pain drugs, in the form of NSAIDs, steroids, gabapentinoids, opioids, and antidepressants, in this study we address the natural compounds belonging to the group of Mexican medicinal plants or “Mexican folk medicine”, used for pain management in Mexico. Our interest in this subject is due to the growing idea that “natural is harmless” and to the large number of side effects exhibited in pharmacotherapy. The objective of this review was to document the scientific evidence about Mexican medicinal plants and their derivatives used for inflammatory and neuropathic pain treatment, as well as the mechanisms of action implicated in their antinociceptive effects, their possible adverse effects, and the main pharmacological aspects of each plant or compound. Our data review suggested that most studies on Mexican medicinal plants have used inflammatory experimental models for testing. The anti-pain properties exerted by medicinal plants lack adverse effects, and their toxicological assays report that they are safe to consume; therefore, more studies should be performed on preclinical neuropathic pain models. Moreover, there is no convincing evidence about the possible mechanisms of action involved in the anti-pain properties exerted by Mexican plants. Therefore, the isolation and pharmacological characterization of these plant derivatives’ compounds will be important in the design of future preclinical studies.

## 1. Introduction

According to the International Association for the Study of Pain (IASP), inflammatory and neuropathic pain are unpleasant and incapacitant conditions that impair the quality of life of those who suffer from that condition. The pathological origins of inflammatory and neuropathic pain are different—inflammatory pain is produced by a lesion in tissue [1], whereas neuropathic pain is a consequence of a lesion or disease affecting the somatosensory system [2,3]. Both inflammatory and neuropathic conditions are commonly represented as chronic pain when the pain lasts or recurs for longer than 3 months [4]. In this way, chronic pain is considered a big problem for the politics of health in developing and developed countries [5]. The prevalence of moderate–severely disabling chronic pain has been estimated to be between 10.4% to 14.3% [6].

A large number of anti-pain drugs for inflammatory and neuropathic conditions have been developed and fully tested in clinical studies [7,8]. The currently pharmacological treatment is usually classified according to the class of pain; moreover, some combinations have been probed to create a synergistic analgesic effect and reduce side effects exhibited by anti-pain drugs [8]. In this regard, NSAIDs such as naproxen, ibuprofen, ketorolac, and some selective COX-2 inhibitors, have side effects on the cardiovascular, gastrointestinal, hepatic, and renal systems [9]. On the other hand, many efforts have been made to prescribe an adequate algorithm for chronic pain treatment. Despite these efforts, the central treatment is focused on opioid pharmacotherapies. Unfortunately, long-term exposure to opioids produces constipation, addictive behavior, tolerance development, and can be a fatal outcome [10]. Moreover, the use of tricyclic antidepressants (TCAs) such as amitriptyline and likewise serotonin and noradrenaline reuptake inhibitors (SNRIs) such as duloxetine and venlafaxine have been associated with somnolence, constipation, dry mouth, and nausea [11]. Furthermore, clinical studies have demonstrated that the prescription of gabapentin or pregabalin is related to somnolence, dizziness, and weight gain; whereas the topical use of lidocaine and capsaicin patches is associated with irritation and local pain [12]. Historically, the wide distribution, use, and acceptance of medicinal plants have been documented in all the regions of Mexico [13,14,15,16,17,18]. In a study carried by Alonso-Castro et al. [13], 28% and 26% of health professionals and physicians, respectively, accepted that they have recommended or prescribed medicinal plants to treat several diseases in their patients. Historically and now, members of the general population have suggested that natural compounds are harmless to the human organism. Because of this, natural products have been used as substitutes for the use of synthetic chemical compounds [19]. However, this is a misconception. It is important to understand that some plants and their derivates might produce side and adverse effects, as well as toxicity and death [20]. In our opinion, the current exploration of the chemical composition, toxicology, dosage, and ethnopharmacology of Mexican herbalism is an urgent area of study for the rational use of traditional medicine. In this respect, there are extensive summaries about the ethnopharmacology of Mexican traditional plants and their derivates, which are popularly employed to treat the most common afflictions. Antibacterial [21] antiparasitic [22], antidiabetic [23] anxiolytic or antidepressant [24,25], anti-cancer [26,27], and cardiovascular [28] effects exhibited by Mexican herbalism products have been demonstrated in preclinical studies. On the other hand, the use of medicinal plants to treat headaches, rheumatic pain, and chronic pain conditions has been documented in Mexican culture [29,30,31]. In this field, there has not been a review of the anti-pain properties of these plants, focusing on the mechanism of actions and adverse effects produced by the most consumed plants in the Mexican population. Accordingly, we propose an extensive review of the preclinical evidence of Mexican medicinal plants and derivative compounds used for inflammatory and neuropathic pain treatment, as well as their main mechanisms of action and reported adverse effects.

## 2. Preclinical Studies of Mexican Medicinal Plants Used in Inflammatory and Neuropathic Pain Treatment

### 2.1. Salvia divinorum Epling and Játiva

**Figure 1:**
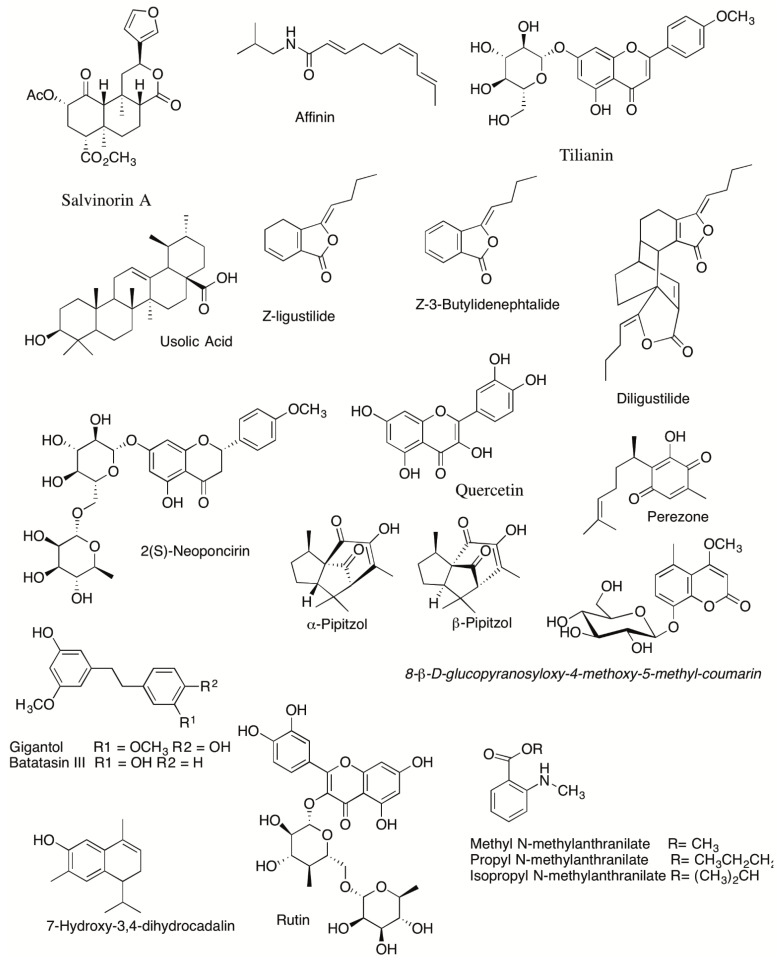

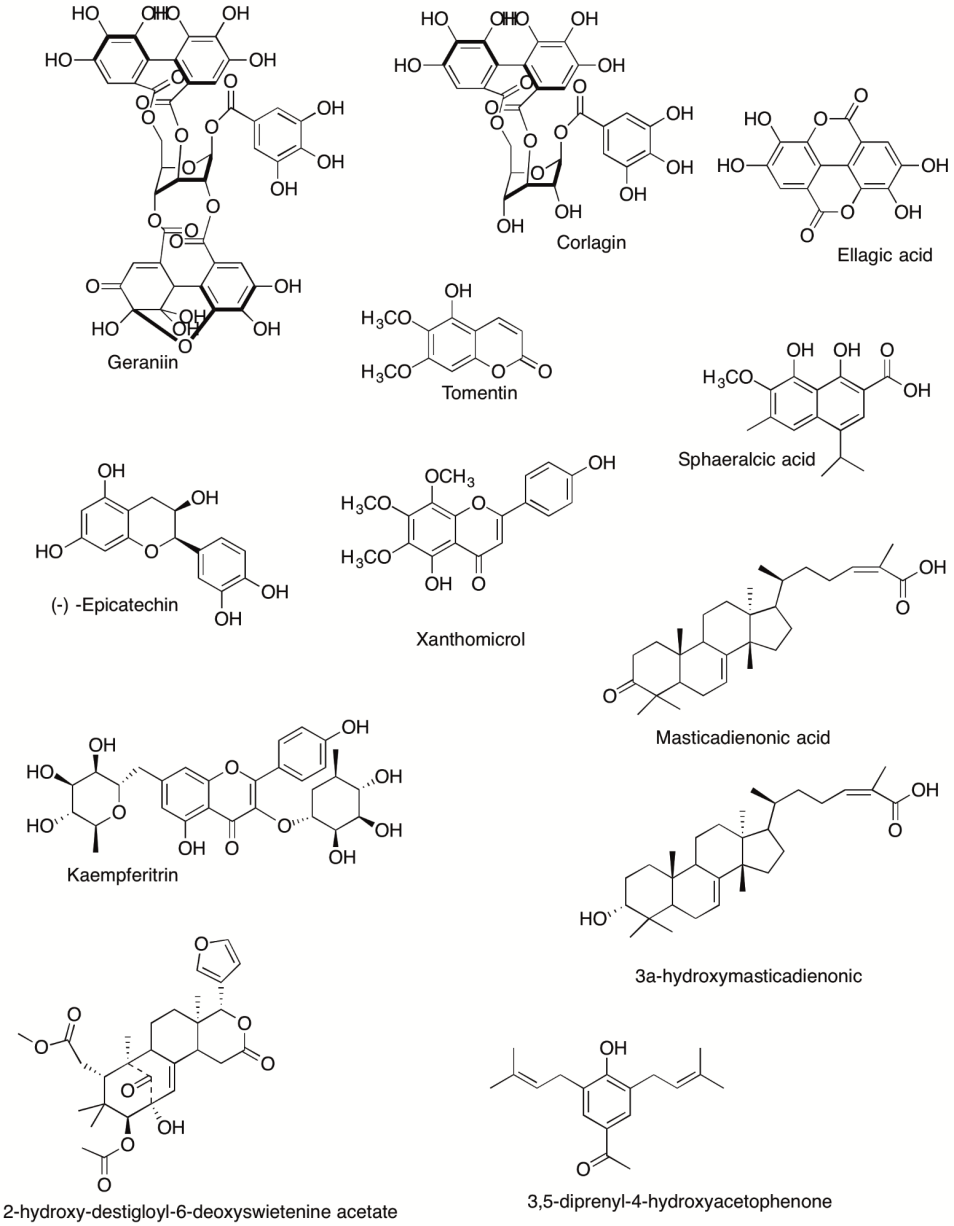
Chemical structures of compounds identified in Mexican plants with anti-inflammatory or antinociceptive effects.

*Salvia divinorum* is a native plant from the Sierra Mazateca region in Oaxaca México. Its name derives from its traditional use by Mazatec people in “mystic” and “spiritual” ceremonies due to its hallucinogenic properties. Since Epling and Játiva-M described the plant [32] based on specimens collected by Hofmann and Wasson (initially named “xka pastora”) [33], the psychopharmacology and effects of this plant belonging to the Lamiaceae family have been studied [34]. *S. divinorum* presents a psychotomimetic selectivity for kappa opioid receptors [35], without serotoninergic actions [36]. The uses of *S. divinorum* extend to anemia, diarrhea, headache, and rheumatism treatments, which have been reported to be practiced by “curanderos mazatecas” or healers [32]. The main constituent of *S. divinorum* is a neoclerodane diterpene, salvinorin A [37]; however, salvinorin B [32] and other constituents such as salvinorins C to J [32,38,39,40], divinatorins A to F [38,40,41], salvinicins A and B [42], and salvidivins A to D [40] have been isolated from *S. divinorum* leaves. In this regard, reports have indicated that under systemic [43,44] and intrathecal [45] administration of salvinorin A (Figure 1), antinociceptive and anti-inflammatory effects were observed [32], whereas anti-neuropathic effects were exhibited with systemic administration of an ethyl acetate extract of *S. divinorum* [46]. Both inflammatory and neuropathic pain models demonstrate that properties of *S. divinorum* are mediated through the activation of kappa opioid receptors [33,34]. More recently, Tlacomulco-Flores et al. [35] developed a preclinical assay with a salvinorin mixture and ethyl acetate extract from *S. divinorum*, suggesting that the antinociceptive effect of extract of salvinorin mixture displayed in the formalin test is due to activation of opioids, whereas the antinociceptive effect in the abdominal contractions test involves opioid and 5-HT_1A_ receptors. Nevertheless, the strong activation of the opioid system is related to hallucinogenic and mystic-type effects. Moreover, cognitive, affective, and perception changes, but not blood pressure, heart rate changes, or adverse effects, were associated with the consumption of salvinorin A [47].

### 2.2. Heliopsis longipes (A. Gray) S.F.Blake

*Heliopsis longipes* from *Asteraceae* family, is a native plant from Sierra de Álvarez and Sierra Gorda in Mexico [36]. Its roots have been used in the Mexican culture for treatment of toothache [37] and as insecticide [38]. The antinociceptive properties of affinin (Figure 1) and *H. longipes* were demonstrated have been several inflammatory pain models in rodents [39,40,41]. Six preclinical assays in mice have documented the antinociceptive behavior produced by the administration of *Heliopsis longipes* or affinin. Concomitantly, experimental assays on brain slices of mice demonstrated that affinin induced the release of GABA [42]. In agreement with these results, Déciga-Campos et al. [43] demonstrated that activation of the GABAergic system, as well as nitric oxide, K^+^ channels, and the opioid and serotonergic systems are responsible for the antinociceptive effects of *H. longipes* and affinin. Moreover, researhcers have also observed the activation of TRPV1 as a mechanism of action of affinin in an inflammatory pain model [44]. Affinin has been described as a stimulant of the nervous system and, paradoxically, has a function as a depressant. Mice submitted to Irwin’s test showed an increased activity and decreased reaction to touch and noise after administration of 1 mg/kg affinin [45]. Moreover, in vitro studies demonstrated the inhibitory effects of *H. longipes* and its main derivate compound, affinin, on CYP3A4, CYP2D6 and CYP1A1/2 [46]. These studies demonstrate that *H. longipes* inhibits the major CYP 450 enzymes involved in the metabolism of 80% of market drugs. Therefore, the ingestion of *H. longipes* in combination with other drugs*,* is likely to increase the concentration of the administrated drugs and subsequently increase the risk of herbal–drug interactions, with adverse clinical consequences.

### 2.3. Artemisia ludoviciana Nutt

*Artemisia ludoviciana* belongs to Asteraceae family and is better known as “estafiate”. It is a medicinal plant that is widely distributed in Michoacán, Querétaro, in the center and the North of México [47]. Since pre-Hispanic times, the use of this plant for treat diarrhea, dysentery, abdominal pain, vomiting, stomachache and muscular spasms has been documented [48]. Moreover, its antihyperglycemic [49] and vasorelaxant [50] properties were demonstrated, as well as antimicrobial effects against *Helicobacter pylori* [51], other bacteria [52,53] and parasites like *Entamoeba histolytica* and *Giardia lamblia* [54]. More recently, its analgesic uses in traditional folk medicine were demonstrated in a preclinical study. *A. ludovicina* decreased nociception in an inflammatory pain model, involving the the participation of the opioid system, without effects on coordination in animals [55]. These results suggested that due to its mechanism of action, *A. ludovicina* could be able to treat inflammatory chronic pain.

### 2.4. Caulerpa mexicana Sonder ex Kützing

The marine algae *Caulerpa Mexicana* belongs to the Cauleparceae family and its distribution is linked to tropical seas. Because of this, it has been found on the coast of Brazil, Florida and Quintana Roo in México [47]. Its antinociceptive, anti-inflammatory and gastroprotective effects have been associated with sulphated polysaccharides from *Caulerpa* [56,57,58]. Some mechanisms of action have been studied, suggesting that its anti-inflammatory actions are related to decreased leukocyte migration [56], whereas that of gastroprotection is mediated by the reduction of oxidative stress and possible prostaglandin actions [57]. Moreover, non toxic effects were observed after the administration of 20 mg/kg of *Caulerpa mexicana,* suggesting an important medical potential.

### 2.5. Agastache mexicana (Kunth) Lint and Epling

*Agastache mexicana* is a folk plant member of the Laminiciae family, which grows in the wild in oak–pine forests of México State, Michoacán, Puebla, Guanajuato, Tlaxcala, Morelos, Veracruz and México City [47,59]. Traditionally known as “toronjil morado”, the common uses of this plant are in the treatment of stomach ache, cough, bile, fever, vomit, nerves and anxiety. However, the main use of *A. mexicana* is to heal “susto” or “espanto”, a cultural disease related with the loss of the soul and characterized by anxiety [60]. Further, the pharmacological properties of its plant have been tested in several preclinical assays, exhibiting effects on spasmolytic activity [61], as well as bronchorelaxant [62] and antihypertensive [63] effects. Several effects on the central nervous system, such as sedation, reduced locomotor activity and central nervous system inhibition, were observed after administration of low doses of *A. mexicana* [64]. Moreover, tilianin, a bioactive compound of *A. Mexicana*, has vasorelaxant [65] and anxiolytic-like activity [66], without toxic effects [67]. On the other hand, ursolic acid and acacetin (Figure 1) are responsible for the antinociceptive properties of this plant [30,68,69], suggesting the participation of cGMP and 5-HT_1A_ receptors. Moreover, the antineuropathic properties of this plant might be revised in the future. Since ursolic acid is the bioactive compound responsible for its antinociceptive effect, there are a reports of anti-hyperalgesia (mechanical and heat) induced by ursolic acid in a chronic constriction injury model; hence, *A. mexicana* may exert a potential effect on neuropathic pain [70].

### 2.6. Ligusticum porteri J.M.Coult. and Rose

*Ligusticum porter*, best known as “chuchupate”, is a plant that grows in the pine-oak forest of the northern Sierra Madre Occidental region [31]. This plant belongs to the Apiaceae family and its traditional uses are related to the alleviation of stomach aches, colic, ulcers, diarrhea, infections, colds and rheumatic joints [71]. The tea from *L. porteri* roots also produce analgesia [72]. Furthermore, the pharmacological effects of this herb have been demonstrated in the treatment of gastric ulcers [73,74,75]. Other pharmacological properties of *L. porter*, such as sedation, anti-spasmolytic [76], anti-inflammatory [77] and antinociceptive properties [78,79], have been observed in preclinical assays. Although this herb is widely consumed as a decoction, there are reports that the chronic administration of the hexane, ethyl acetate and methanol extracts of *L. porteri* produce toxicity [80]. Concomitantly, slight effects of acute toxicity were observed in mice, whereas a weak LC50 value (ranging from 436 to 778 μg/mL) was observed in the brine shrimp lethality assay [81]. Furthermore, a phytochemical assay demonstrated the presence of flavonoids, phenols/tannins, triterpenoids/steroids and traces of alkaloids as the main constituents of *L. porteri* [80]. Phthalides are the major secondary metabolites. Relatedly, Z-ligustilide, Z-butylidenephtalide and diligustilide (Figure 1) are the main bioactive compounds of this plant, which is responsible for the major pharmacological effects of *L. porteri* [82]. However, the mechanisms of action leading to its antinociceptive properties remain unclear.

### 2.7. Clinopodium mexicanum Benth Govaerts

*Clinopodium mexicanum*, named “toronjil de monte”, is a plant of the Laminiceae family. The common uses of this plant are to induce sleep and analgesia [83]. There is little information on the pharmacological effects of this medicinal plant. However, some reports indicate that 2(S)-neoponcirin (Figure 1) is the compound responsible for the anxiolityc and antinociceptive effects of *C. mexicanum*. Regarding this, the activation of the GABAergic system explains the anxiolytic or depressant effect of this plant [84]. On the other hand, the flavanone glycosides neoponcirin, poncirin and isonaringenin have been identified as the main constituents of *C. mexicanum* [83].

### 2.8. Tilia americana var Mexicana (Schltdl.) Hardin

*Tilia* is a tree belong to the Malvaceae family, which is recognized in the Mexican population for its uses in the treatment of sleep disorders or anxiety [85,86]. Flower infusions are the most common form of ingestion of this plant. Its distribution is wide, from Nuevo Leon and Tamaulipas to Oaxaca state, with a marked predominance in Michoacán State [47]. The anxiolytic effects produced by the ingestion of inflorescences of *Tilia* var mexicana have been attributed to kaempferol-3,7-O-dirhamnoside (kaempferitrin), quercetin-3-pentosylhexoside, kaempferol-3-pentosylhexoside, quercetin-3-O-glucoside (isoquercitrin), kaempferol-3-O-glucoside (astragalin), quercetin-3-O-rhamnoside (quercitrin), kaempferol-3-O-rhamnoside, kaempferol-3-O-(6-p-coumaroyl)-glucoside (tiliroside), quercetin-3,7-O-dirhamnoside, quercetin-3-O-rutinoside (rutin), quercetin-3-pentoside, quercetin-malonylhexoside, which were detected in *Tilia* flowers and bracts from three different regions of Mexico [87,88]. Preclinical pharmacological studies indicate that flowers of *Tilia americana* var mexicana exhibit anxiolytic, sedative [89] and anticonvulsant effects [90], as well as potential properties in the management of stroke [91]. In addition, preclinical evidence suggests the use of *Tilia* for pain treatment [92], suggesting that quercetin (Figure 1) is responsible for its pharmacological activity through the activation of 5-HT_1A_ receptors.

### 2.9. Acourtia thurberi (A. Gray) Reveal and R. M. King

*Acourtia thurberi*, “Matarique” or “Matarique morado”, belongs to the family Asteraceae. This perennial herb is a medicinal plant with lavender or purple flowers and is found in the northern Sierra Madre Occidental and the mountains of southern Arizona and New Mexico [93]. Normally, it is consumed in a tea containing its roots, for the treatment of kidney disease, diabetes and back pain associated with the kidneys [31]. *A. thurberi* has antihyperglycemic [94] and antinociceptive effects [95]. In the latter study, the pharmacological effects were attributed to the presence of perezone, *α*-pipitzol, *β*-pipitzol and 8-β-D-glucopyranosyloxy-4-methoxy-5-methyl-coumarin (Figure 1), which were isolated from the roots of *A. thurberi.*

### 2.10. Cyrtopodium macrobulbon (Lex.) G.A. Romero and Carnevali

*Cyrtopodium macrobulbon*, or “cañaveral”, is a folk plant that has not been widely studied. However, this member of the Orchidaceae family is currently used for the treatment of a painful urinary condition commonly named “mal de orin” [96]. In experimental assays, Morales-Sanchez et al. [96] demonstrated that visceral pain in mice is reduced through the systemic administration of organic and aqueous extracts of *C. macrobulbon*. In the same study, some of the compounds detected in *C. macrobulbom* extract were *n*-hexacosyl-*trans*-*p*-coumarate, *n*-octacosyl-*trans*-*p*-coumarate, *n*-triacontyl-*trans*-*p*-coumarate, 4-methoxy-benzyl alcohol, 4-hydroxybenzaldehyde, 1,5,7-trimethoxy-9,10-dihydrophenanthrene-2,6-diol, confusarin, gigantol, batatasin III and ephemeranthol B. The antinociceptive effect was attributed to the presence of gigantol and betatasin III (Figure 1).

### 2.11. Ternstroemia sylvatica Schltdl. and Cham

Since ancestral times, decoctions of *Ternstroemia sylvatica* fruits, best known as “flor de tila” or “capulincillo”, have been used to treat anxiety disorders [97]. This folk medicinal plant from the family Theaceae is distributed in Ciudad de México, Hidalgo, San Luis Potosi, Chiapas, Querétaro, Veracruz Tamaulipas and Sinaloa [47,98,99]. Pharmacological studies of *T. sylvatica* fruits have demonstrated that the common anxiolytic uses of this plant are due to its sedative properties [100,101]. In addition, its traditional uses are supported by its other actions, such as anti-inflammatory and analgesic effects displayed in a murine model, suggesting that its actions are mediated by the activation of antioxidant mechanisms [99]. Furthermore, Balderas-López et al. [100] demonstrated that triterpene glycoside 28-*O*-[β-l-6-rhamnopyranosyl]-R_1_-barrigenol isolated from aqueous extracts of seeds of *T. sylvariva* fruits is the bioactive compound responsible for its sedative effects, but this also presents toxic and lethal effects.

### 2.12. Conyza filaginoides (D.C.) Hieron

*Conyza filaginoides* or “simonillo” is a medicinal plant belong to the Asteraceae family, which is employed in Mexican culture to treat stomach ailments [102]. Lately, antihyperglycemic uses have been suggested [23,103]. In México, this plant is widely commercialized and its presence was recently reported in geographic areas such as Querétaro, Nuevo León and Oaxaca [47]. The presumable antiparasitic activity of this traditional plant was tested in in vitro assays; however, none of the constituents of *C. filaginoides* were able to show antiprotozoal activity against *Giardia lamblia* or *Entamoeba histolytica* [104]. Regarding the pharmacological actions of constituents from *C. filaginoides*, a relaxing effect on the smoot muscle in the ileum rat was related with flavonoids, sterol, sesquiterpenoid and triterpenoids [105]. Furthermore, antinociceptive effects of organic extracts of *C. filaginoides* were demonstrated in normo- and hyperglycemic mice, suggesting that this folk plant can be used in inflammatory and neuropathic pain treatment. Moreover, the authors suggested that rutoside, quercetin-3-O-rutinoside, also commonly called “rutin” (Figure 1), induces antinociceptive effects, which is prevented by the administration of flumazenil, bicuculline or naltrexone in a formalin test. These results suggest that rutoside, one of the main constituents of *Conyza filaginoides*, is the compound responsible for its antinociceptive effects, suggesting the activation of the GABAergic and opioid systems [103].

### 2.13. Choisya ternata Kunth

*Choisya ternata* is an ornamental plant with leaves and white flowers which resemble an orange tree. Due to this, its English name is “Mexican orange”; however, in Mexican culture can be identified by other popular namesm such as “flor de clavo”, “clavillo” or “clavo de olor” [106]. As a peculiarity, this plant is a member of the Rutaceae family and is native to the central and southern mountains of México [85]. The most important folk uses of infused leaves from *Choisya ternata* are related to its antispasmodic and stimulative properties. Moreover, pharmacological studies have suggested antidepressant, anxiolytic and antinociceptive activities of *C. ternata* and its derivates [107,108]. In line with these activities, antinociceptive effects have been associated with compounds such as isopropyl, methyl and propyl N-methylanthranilates (Figure 1), obtained from the plant. Apparently, these bioactive compounds mediate the activation of K^+^_ATP_ channels, as well as serotonergic, adrenergic and nitrergic pathways [109] to produce antinociceptive effects. Moreover, the anti-inflammatory and antinociceptive effects of essential oils from *C. ternata* have been linked to the inhibition of nitric oxide, TNF-α and IL-1-β [110].

### 2.14. Mimosa albida Humb. and Bonpl. ex Willd

*Mimosa albida* from the Fabaceae family is a common undergrowth, geographically distributed in all of México [47,85]. This traditional medicinal plant is known as “uña de gato” and decoctions of its leaves are ingested to treat gastritis, cancer, diabetes, diarrhea and wounds [16]. Pharmacological studies have suggested that the aqueous root extract of *M. albida* had an antinociceptive effect on an inflammatory model. In the same study, the aqueous extract did not show anxiolytic effects; however, motor activity and coordination in mice were affected by low doses of *M. albida* extract. No mortality was observed in an acute toxicity test [111].

### 2.15. Heterotheca inuloides Cass

“Arnica Mexicana” is the common folk name associated with *Heterotheca inuloides*. This plant, belonging to the Asteraceae family, is widely used in flower tea form to treat bruises, rheumatism, inflammation, gastric ulcers, bile duct diseases, cancer and lung diseases [112]. The anti-inflammatory effect of *Heterotheca inuloides*, as well as antinociceptive properties, have been demonstrated in preclinical models [113]. The inhibition of COX has been identified as part of its mechanism of action [114], along with the peripheral activation of 5-HT1 receptors [115]. In agreement with these effects, Rocha-González et al. [116] demonstrated that the antineuropathic actions of 7-hydroxy-3,4-dihydrocadalin (Figure 1) involved the activation of serotonergic and opioid receptors, as well as the activation of guanylyl cyclase. An antioxidant effect was also observed. On the other hand, in vitro assays demonstrated the bactericidal, antiparasiti, and cytotoxic activity of 7-hydroxy-3,4-dihydrocadalin, supporting its traditional uses in Mexican culture [117,118,119]. In the last two decades, the information on the pharmacology, toxicology and chemical composition of this plant has increased. Twenty compounds were isolated from dried flowers of *H. inuloides*, demonstrating that the major constituent is the sesquiterpenoid 7-hydroxy-3,4-dihydrocadalin [119,120]. In the same study, the authors suggested that 7-hydroxy-3,4-dihydrocadalin can be linked to alterations in body weight, hepatoxicity, nephrotoxicity and death at very high doses [119].

### 2.16. Calea Zacatechichi Schltdl

*Calea zacatechichi* is an oneirogenic plant belonging to the Asteraceae family. This folk plant grows in savannahs and canyons of Mexico, especially in Oaxaca, where is consumed as an infusion of the roots, leaves and stem of the plant. *C. zacatechichi* is popularly named “hoja madre”, “zacate de perro” or “pasto amargo” among Chontal people from Oaxaca. They use the infusions to induce sleep, divinatory dreams, analgesia and to exert antipsychotic effects [121]. The ethnopharmacology of this plant has reported anti-inflammatory [122] and antihyperglycemic effects [123], as well as the antileishmanial activity of germacranolides [124]. Moreover, the analgesic uses of this plant have been demonstrated in several inflammatory pain models [125,126]. However, the bioactive compounds which induce its anti-inflammatory and antinociceptive effects are yet unknown, and the antinociceptive pathways activated by *C. zacatechichi* are still unclear. Moreover, some side effects linked to this plant include signs of somnolence and sleep, salivation, ataxia, retching and occasional vomiting. On the other hand, healthy volunteers administered with *C. zacatechichi* reported an increase in the superficial stages of sleep, associated with an increase in hypnagogic imagery [121].

### 2.17. Geranium bellum Rose

*Geramium bellum* Rose is a traditional plant commonly used to treat fever, pain and gastrointestinal disorders. In the local market is best known as “pata de león”; however, the origin of this plant can be inferred from its perennial growth in the mountains of the Hidalgo State of México [127]. The genus *Geramium* of the family Geraniaceae has been widely studied; however, *G. bellum* and its pharmacological effects have not been extensively studied. Concerning the phytochemical composition of this plant, the literature reports that corilagin, gallic acid, methyl gallate, methyl brevifolincarboxylate, quercetin, quercetin 3-O-β-D-glucopyranoside, quercetin 3-O-β-D-[6″-O-galloyl)glucopyranoside, kaempferol, β-sitosterol 3-O-β-D-glucopyranoside, beta-sitosterol and kaempferol 3-O-β-D-glucopyranoside are the major bioactive constituents of *G. bellum* [128]. The chemical description of these compounds is important in order to give an idea about the pharmacological properties of this folk plant. In line with this, methyl brevifolincarboxylate, ethylbrevifolin carboxylate and butylbrevifolin carboxylate compounds are responsible for the antiparasitic activity of this plant [129]. More recently, quercetin, geraniin, corilagin and ellagic acid (Figure 1) isolated from an acetone-aqueous extract of *G. bellum* displayed antinociceptive and anti-inflammatory effects in a murine model. Nevertheless, the mechanism of action underlying its antinociceptive effect was not studied [127].

### 2.18. Piper auritum Kunth

Since ancient times, *Piper auritum* has been named “hierba santa” in the indigenous Mexican culture. This plant belongs to the Piperaceae family and its ingestion is in a tea form prepared using fresh leaves to treat several afflictions, such as sore throat, dermatological illness, diabetes and wounds [130,131]. *P. auritum* is consumed during the gestation stage to improve digestion and alleviate flatulence; however, one of the precautions is that the effects of this plant might be abortive [132]. Phytochemical studies of *P. auritum* have demonstrated that more than 30 compounds were identified in essential oil from *P. auritum*; however, the main bioactive compound was safrole (87%). This compound could be responsible for the antiparasitic activity of this plant [133]. On the other hand, *Piper auritum* has demonstrated positive effects on diabetes, cholesterol and triglycerides [134,135]. Moreover, the administration of *P. auritum* did not reduce carrageenan-induced paw edema in rats [136]. This result is important in order to clarify the pharmacological activities related to the traditional uses of *P. auritum* because some cultures employ the decoction of its leaves to treat headaches and to induce local anesthesia [131].

### 2.19. Sphaeralcea angustifolia (Cav.) G. Don

*Sphaeralcea angustifolia*, popularly known as “hierba del negro” “hierba del golpe” or “vara de San José”, is a member of the Malvaceae family. In México, traditional medicine indicates the topical use of aerial parts of this plant for bruises and swelling [137]. The pharmacological properties of this plant have been developed in preclinical and clinical studies. The anti-inflammatory activity of chloroform extracts from *S. angustifolia* was demonstrated in a carrageenan-induced paw edema test in mice. However, systemic administration of higher doses of hexane extracts of S. angustifolia displayed toxicity and lethal effects [136]. Further, a clinical trial in patients with hand osteoarthritis administered over 4 weeks with a gel containing 1% of *S. angustifolia* extract, demonstrated efficacy and tolerability; however, the treatment was not different to the patients undergoing diclofenac treatment [138]. Tomentin and sphaeralcic acid (Figure 1) were identified as the bioactive compounds responsible for the anti-inflammatory effects of *S. angustifolia* [137]. Hence, preclinical, and clinical studies support the correct folk uses of *S. angustifolia.*

### 2.20. Acacia farnesiana (Willd.) Kuntze

*Acacia farnesiana* [47,85] or “huizache” is one of the shrubs most common in arid and semiarid regions throughout México. This plant belonging to the Fabaceae family is characterized by the production of a pod that is rich in fiber, protein, nitrogenated elements and tannins, which are the main source of nutrients for wild sheep [139]. The common medicinal uses of huizache are to treat diarrhea, dysentery, tuberculosis and indigestion. Phytochemical characterization of this plant revelated that 22E-stimasta-5,22-dien-3β-ol, 22E-stimasta-5,22-dien-3β-ol, 22E-stimasta-5,22-dien-3β-acetyl, 22E-stimasta-5,22-dien-3β-acetyl, tetracosanoic acid (2S)-2, 3-dihydroxypropyl ester, tetracosanoic acid (2*S*)-2, 3-dihydroxypropyl ester, stigmasta-5,22-dien-3β-O-D-glucopyranoside, stigmasta-5,22-dien-3β-O-D-glucopyranoside, stigmasta-5,22-dien-3β-O-D-tetraacetylglucopyranoside, stigmasta-5,22-dien-3β-O-D-tetraacetylglucopyranoside, methyl gallate, methyl 3,4,5-triacetyloxybenzoate, methyl 3,4,5-triacetyloxybenzoate, gallic acid, (2S)-naringenin 7-O-β-D-glucopyranoside, (2S) -naringenin 7-O-β-D-glucopyranoside and pinitol are compounds present in hexanic-chloroformic and methanolic extracts of *A. farnesiana* [140]. Furthermore, the presence acasiane A, acasiane B, farnesirane A and farnesirane B was reported by Lin et al. [141]. Relaxant and anti-inflammatory effects have been observed in a glucosidal fraction of the pods of this plant [142]. On the other hand, the antinociceptive effects of this plant were studied in an inflammatory model. The methanol extract of *A. farnesiana* reduced the paw edema induced by carrageenan; however, the hexane and chloroform extracts produced death in the animals [136]. This antinociceptive effect might be explained by the fact that chloroformic, hexanic and ketonic extracts from *A. farnesiana* induce anti-inflammatory effects through the inhibition of prostaglandins and interleukins such as IL-1β, TNF-α, and IL-6 [143].

### 2.21. Rubus coriifolius Liebm

*Robus coriifolius* is a plant belonging to the Rosaceae family, which growths in a wild form in Michoacán, Veracruz, Morelos and Chiapas. This plant has a red-black fruit and because of this it is named “zarzamora silvestre”. Local people use a decoction of this plant to treat diarrhea and dysentery [144]. (-)-Epicatechin, (+)-catechin, hyperin, nigaichigoside F1, β-sitosterol 3-O-β-d-glucopyranoside, gallic acid and ellagic acid are some of the chemical bioactive compounds of this plant [145]. Moreover, the evidence suggests that antiparasitic effects exerted by *R. coriifolius* are mediated by (-)-epicatechin [146]. These results support the ethnopharmacology and traditional uses of *R. coriifolius*. In line with preclinical studies, its anti-inflammatory properties have been demonstrated in an inflammatory pain model induced by carrageenan [136]. No toxicity was observed at anti-inflammatory doses. In addition, these data are supported by the genotoxicity and subacute toxicity testing of ethanolic extracts of *R. coriifolius* [147]. Since its major bioactive compound is (-)-epicatechin (Figure 1), which displays anti-inflammatory and anti-neuropathic effects [148], *R. coriifolius* might be explored in other experimental models of pain.

### 2.22. Oenothera rosea L’Hér. ex Aiton

*Oenothera rosea* is a native plant also named “hierba del golpe”, which belongs to the Onagraceae family. It has been commercialized mainly to treat bruising and swelling and its geographic disposition is wide, throughout all of Mexico [47]. The pharmacological effects of this plant have been demonstrated in an inflammatory model of colonic damage [149] and a gastric cancer model [150]. Moreover, in carrageenan-induced inflammatory pain in rats, a methanol extract of *O. rosea* was able to reduce the paw edema for almost 7 h. In the same study, non-lethal effects were observed in the rats [136]. Moreover, the analgesic effect of *O. rosea* was displayed by the increased dose of ethanol or ethyl acetate extract (50–200 mg/kg). Oral administration of both extracts increased the latency response on the hot plate test and decreased the number of writhings induced by the acetic acid test [151].

### 2.23. Chamaedora tepejilote Oerst

*Chameadora tepejilote*, commonly named “tepejilote” or “palma pacaya”, is a medicinal plant that is widely distributed, mainly in Oaxaca, Chiapas, Veracruz and Tabasco. It is a member of the Arecaceae family. It has been used in folk medicine to treat illnesses or afflictions related to respiratory functions. Its traditional uses are supported by pharmacological studies in experimental tuberculosis, in which ursolic and oleanolic acid, as well as squalene and farnesol isolated from *C. tepejilote*, displayed antimicrobial activity against *Mycobacterium tuberculosis* [152,153,154]. On the other hand, preclinical studies indicate that aqueous and methanol extracts from *C. tepejilote* have anti-inflammatory properties, whereas the hexane extract of this plant resulted in the death of animals [136]. In this regard, the phytochemical composition of tepejilote indicates the presence of bioactive compounds such as ursolic acid, which have an important therapeutic potential to treat pain [68,69]. In the future, ursolic acid from “tepejilote” might be studied in a neuropathic pain model, due to its antinociceptive effects demonstrated on several inflammatory pain models.

### 2.24. Astianthus viminalis (Kunth) Baill

*Astianthus viminalis* is a folk plant called “Azuchil” by residents of southern Mexico [47]. This plant belongs to the Bignoniaceae family, and its phytochemical composition was studied for the first time by Alvarez et al. [25]. Antimicrobial properties have been proposed for cinnamic and p-methoxycinn acid, the iridoid glucoside campenoside and 5-hydroxycampenoside compounds. In the same study, other bioactive compounds, such as ursolic and oleanolic acids, were detected. Moreover, 3β,19α-dihydroxyurs-12,20(21)-diene-28-oic acid, a derivate from this plant, showed an anti-hyperglycemic effect in an experimental model of diabetes [155]. This study supported and addressed the antidiabetic uses of this plant in Mexican folk medicine. To our best knowledge, *Astianthus viminalis* is not used to treat inflammatory or neuropathic pain, although there is evidence on the anti-inflammatory effects of the methanol extract of this plant on carrageenan-induced paw edema in rats [136].

### 2.25. Brickellia veronicaefolia Kunth DC

*Brickellia veronicaefolia* belongs to the family Asteraceae and is commonly named “hierba dorada”. Its geographic distribution ranges from the oak–pine forests of Coahuila to Oaxaca. In accordance with its folk uses, this plant has antihyperglycemic properties [23]. In this regard, the hexane extract and the bioactive compound 5,7,3′-trihydroxy-3,6,4′-trimethoxyflavone have hypoglycemic [156,157] and antioxidant activity [158]. In addition, other compounds such as benzyl 2,6-dimethoxybenzoate, 2-hydroxybenzyl 2′-methoxybenzoate, chamazulene, beta-caryophyllene, germacrene D, bicyclogermacrene, β-eudesmol, [159] 1,2-bis-O-(2-methoxybenzoyl)-β-d-glucopyranoside, 3-(β-glucopyranosyloxy)benzyl 2,6-dimethoxybenzoate and 3-hydroxybenzyl 2,6-dimethoxybenzoate, together with the known compounds taraxasteryl acetate, 4-allyl-2-methyloxyphenyl-beta-glucopyranoside (5), 2-hydroxy-6-methoxybenzoic acid, 2-methoxybenzoic acid, 2-methoxybenzyl 2-hydroxybenzoate, 3-methoxybenzyl 2-hydroxy-6-methoxybenzoate, benzyl 2-hydroxy-6-methoxybenzoate, benzyl 2,3,6-trimethoxybenzoate, benzyl 2-hydroxy-3,6-dimethoxybenzoate and 3-methoxybenzyl 2,6-dimethoxybenzoate [160], have been isolated from *B. veronicaefolia*. On the other hand, the relaxant [160] and antinociceptive properties [79] of this plant were demonstrated in preclinical assays. However, the mechanism of its antinociceptive effects have not yet been studied.

### 2.26. Brickellia paniculata (Mill.) B.L.Rob

Since ancient times, *Brickellia paniculata*, a member of the Asteraceae family, has been used to treat stomach pain, colic and diarrhea in southern Mexico [144]. Xanthomicrol and 3-α-angeloyloxy-2α-hydroxy-13,14Z-dehydrocativic acid are its bioactive compounds, which are also characterized as relaxant agents. This explains the popular folk uses of *B. paniculate* leaves to treat gastrointestinal spams [161,162]. Furthermore, preclinical evidence addressed the anti-inflammatory properties of methanol extracts of *B. peniculata* in carrageenan-induced paw edema [136]. To our knowledge, there have been no more preclinical studies in neuropathic or inflammatory pain to indicate the antinociceptive properties of *B. peniculata*. However, the isolated compound xanthomicol (Figure 1) can block the voltage-operated calcium channel; hence, this compound may show therapeutic potential to treat pain [163].

### 2.27. Justicia spicigera Schltdl

“Muicle”, “micle” and “moyottli” are some names used by the people in Michoacán, Tabasco, Nayarit, Jalisco, San Luis Potosi, Chiapas, Morelos, Tlaxcala, Veracruz and Yucatan state to identify the *Justicia spicigera* plant. It is a plant belong to the Acanthaceae family and has been used since Aztec times as an infusion of the leaves, branches, and flowers for consumption as a common drink throughout the day. Its infusions are indicated to treat inflammation, anemias, leukemias, pulmonary tuberculosis, diarrhea, hemorrhoids, parasites, rheumatism, arthritis, bone disease and diseases of the eye [164]. Furthermore, its properties have been used since pre-Hispanic times to obtain indigo dye for paintm food, baskets, crafts and clothes. Concerning its ethnopharmacology, preclinical assays have demonstrated the anticonvulsant properties induced by aqueous extracts of *J. spicigera* and its derivate kaempferitrin [165], as well as antidepressant [166] and anxiolytic-like effects [167]. In the same way, the antidiabetic, [168] antitumor, immunomodulatory [169], anti-inflammatory [136] and antiparasitic [170] properties of this plant have been demonstrated. Pertaining to the topic of this review, *J. spicigera* has reduced nociception in several inflammatory pain models [171], suggesting that its antinociceptive effect can be attributed to kaempferitrin (Figure 1), which in turn induces antispasmodic effects through the activation of 5-HT_1A_ and opioid receptors [172,173]. As a recapitulation, the pharmacological studies support the ancient use of *J. spicigera* to alleviate painful conditions. However, it is important to consider that hexane and chloroform extracts of this plant displayed mortality in mice administrated with 400 mg/kg of those extracts.

### 2.28. Lantana hispida Kunth

*Lantana hispida*, popularly known as “morita negra”, is a small shrub or herb that grows in cleared places. This plant from the family Verbenaceae is widely distributed in México, with recent observations in Baja California Sur, San Luis Potosi and Chiapas States [47]. The popular form of consumption of this plant is on the infusion of the fruits and leaves to alleviate coughs, whereas bathing with this plant in water is commonly used in the “Tajin” area as a protection against “mal de viento” or “mal aire” in children [174]. The phytopharmacological characterization of *L. hispida* and its compounds has been described to treat tuberculosis [153,175]. 3-acetoxy-22-(2′-methyl-2Z-butenyloxy)-12-oleanen-28-oic acid, 3-hydroxy-22 beta-(2′-methyl-2Z-butenoyloxy)-12-oleanen-28-oic acid (reduced lantadene A), oleanolic acid and ursolic acid have been described in the phytochemical composition of this plant [154]. In line with this review, systemic administration of butanol extract from *L. hispida* demonstrated an anti-inflammatory effect on carrageenan-induced paw edema [136]. Moreover, antinociceptive properties of the *Lantana* genus, as in *Lantana trifolia*, have shown anti-inflammatory and analgesic effects on carrageenan- and histamine-induced paw edema, as well as in hot plate and tail-flick thermal tests [176,177]. Because of this, *L. hispida* should be considered for evaluation in several neuropathic and inflammatory pain models, and its isolated compounds could also be tested.

### 2.29. Pittocaulon bombycophole (Bullock), velatum (Greenm), praecox (Cav.) and hintonii H.Rob. and Brettell

The *Pittocaulon* genus is endemic to Mexico and it grows in the form of strange shrubs and small trees in dry and semiarid parts of central and southern Mexico. Its common name in popular culture is “palo loco”. The genus *Pittocaulon* belongs to the Asteraceae family and includes five species—*P. praecox*, *P. velatum*, *P. bombycophole*, *P. hintonii* and *P. filare* [178,179]. The historical folk use of this plant is to treat rheumatism and antiinflammatory ailments [180]. The chemical composition of *Pittocaulon* has been described, and pyrrolizidine alkaloids were identified in the five species of its genus [181,182], whereas sesquiterpenois with eremophilane skeletons were found in *P. praecox, P. bombycophole*, *P. velatum* and *P. filare* species [178]. Regarding the traditional uses of this plant as medicine, *P. praecox, P. velatum, P. bombycophole* and *P. hintonii* species demonstrated antibacterial and antifungal activity [182]. On the other hand, extracts of *P. velatum* and a methanolic extract of *P. bombycophole* inhibited 12-O-tetradecanoylphorbol 13-acetate (TAP)-induced ear edema, presumably through an antioxidant effect, as demonstrated in the thiobarbituric acid reactive substances (TBARS) assay [181]. In the same study, the carrageenan test—a model characterized by inflammation and hyperalgesia—was used to test the anti-inflammatory effect of dichloromethane extracts of the roots of different species of *Pittocaulon* (100 mg/kg). The results did not show a significant inhibition of paw edema. Moreover, sesquiterpenoids present in *Pittocaulon filare* inhibited the neutrophil infiltration in ear edema [178].

### 2.30. Amphipterygium adstringens Standl

*Amphipterygium adstringens* belongs to the Anacardaceae family, which is widely known due to its beneficial effects on circulatory problems, ulcers and gastric infections including *H. pylori*. Tea made from the tree bark is the most common form of consumption of this plant, commercialized as “Cuachalalate”. This plant contains anacardic acids, triterpenoids and sterols as major components. In a study, eight compounds were isolated from this folk plant: anacardic acid, 6-[16′Z-nonadecenyl]-salicylic acid, 6-[8′Z-pentadecenyl]-salicylic acid 6-nonadecenyl-salicylic acid, 6-pentadecyl-salicylic acid, masticadienonic acid, 3α-hydroxymasticadienonic acid, 3-epi-oleanolic acid and β-sitosterol [183]. Some of these compounds exhibited antibacterial activity, whereas the alcoholic extract of *A. adstringens* was able to reduce colitis ulcerative in a preclinical mouse model [184] and the alcoholic extract produced gastroprotection in rats [185]. Moreover, antinociceptive properties of *A. adstringens* have been studied in acetic acid-induced writhing; however, the extract of the plant did not reduce the nociception [79]. Furthermore, aqueous and hexane extracts from *A. adstringens* reduced ear and paw edemas in mice, suggesting that masticadienonic and 3*α*-hydroxymasticadienonic compounds (Figure 1) produce the inhibition of nitrites as a possible mechanism of action [186]. Likewise, the anti-inflammatory potential of this plant was addressed by Arrieta et al. [187], highlighting nitric oxide inhibition, which has a fundamental role in inflammatory pain treatment. In summary, preclinical assays have demonstrated the anti-inflammatory but not the antinociceptive effect of *A. adstringens*.

### 2.31. Gnaphalium *sp.*

At least 26 species of the *Gnaphalium* genus are popularly called “Gordolobo”, belonging to the Asteraceae family. These plants are widely distributed in Mexico, with a strong presence in the central states of Mexico. In folk medicine its inflorescences are used in a tea form to treat respiratory ailments like asthma, flu, cough, expectorating, fever and bronchial infections [188]; nevertheless, its antibacterial activity is the most characterized effect in preclinical assays. In this respect, Gnaphalium *oxyphyllum* var *oxyphyllum (DC.) Kirp, G. liebmannii* var*. monticola* (McVaugh) D.L.Nash, *G. viscosum* (Kunth) and *G. americanum* (Mill) are Mexican species that have demonstrated great potential as antibacterial agents [189,190]. The phytochemical composition of the *Gnaphalium* genus includes flavonoids, sesquiterpenes, diterpenes, triterpenoids, phytosterols, anthraquinones, acetylenic compounds and carotenoids [188,190]. Concerning toxic effects, *Gnaphalium* sp. was not toxic to *Artemia salina*, a lethality assay preliminary to toxic tests; whereas the Ames assay demonstrated the mutagenic potential of this plant [81]. The antinociceptive effect of *Gnaphalium* sp. was investigated by Déciga-Campos et al. [79]; however, the dichloromethane-methanol extract of this plant did not produce an antinociceptive effect in an acid acetic-induced writhing test in mice. Nevertheless, the anti-inflammatory properties of *Gnaphalium affine* D. Don, used in traditional Chinese medicine, were demonstrated in two inflammatory pain models, carrageenan-induced paw edema and collagen- induced arthritis [191].

### 2.32. Swietenia humilis Zucc

*Swietenia humilis,* belonging to the *Meliaceae* family, is a medium-size tree grown in tropical areas in Mexico. This tree is also known as “Zopilote” or “caobilla”, and produces a seed that is used to treat diabetes type 2 [23]. Due to its folk uses, preclinical assays of this plant have been focused on diabetes type 2 and metabolic syndrome, finding that it produced antihyperglycemic, hypoglycemic and hypolipidemic effects, suggesting the participation of K_ATP_ channels, insulin secretion and the modulation of 5-HT_2_ receptors [192,193]. Limonoids (a form of triterpenes) are the bioactive compounds characteristic of the Meliaceae family, and therefore are responsible for the mechanisms of action of *Swietenia humilis* [194]. The antinociceptive properties of aqueous extracts of *Swietenia humilis* and the mexicanolide 2-hydroxy-destigloyl-6-deoxyswietenine acetate (Figure 1) were investigated in formalin-induced hyperalgesia in diabetic rats. The data suggested that its anti-hyperalgesic effect is mediated by the activation of nitric oxide and GABAergic, opioidergic and serotonergic (5-HT_2A/C_) pathways, as well as activation of guanylyl cyclase and K_ATP_ channels [195]. Although the mechanism of action of this plant has been studied, to date there have been no more studies on its effect on neuropathic or inflammatory conditions. We suggest thatlimonoids from *Swietenia humillis* should be evaluated in inflammatory pain models.

### 2.33. Ageratina pichinchensis (Kunth) R. King and H. Rob.

*Ageratina pichinchensis* or “axihuitl” is an endemic plant from Morelos State, which belongs to the Asteraceae family. This plant is also known by the scientific names *Eupatorium aschembornianum* (Sch.) and *Eupatorium bustamentum* DC. [93,196]. This medicinal plant has been used for the treatment of skin wounds. Its pharmacological effects were demonstrated in preclinical assays of experimental wounds in streptozotocin-induced diabetic mice and ethanol-induced gastric ulcers [197,198]. 7-*O*-(β-d-glucopyranosyl)-galactin and 3,5-diprenyl-4-hydroxyacetophenone were identified as responsible for the pharmacological properties of this plant. No genotoxic effects were observed under the administration of aqueous and hexane-ethyl acetate extracts of *A. pichinchensis*. Moreover, unpigmented hexane-ethyl acetate extract at 5% was evaluated over 11 days in patients with stomatitis, showing that *A. pichinchensis* reduces the size of buccal lesions and pain scores, with an effectivity of 100% at the end of the study [199]. Clinical uses of this folk plant have been extended to onychomycosis, vulvovaginal candidiasis [200], diabetic foot ulcers [201] and tinea pedis [202]. Despite several clinical studies, there is only one preclinical study about the antinociceptive and anti-neuropathic properties of this plant. In that study, oral administration of 100 and 556 mg/kg of 3,5-diprenyl-4-hydroxyacetophenone (Figure 1) from *A. pichinchensis* reduced nociception in the carrageenan model and spinal nerve ligated rats; however, the mechanisms of action of these effects were not demonstrated [198]. In the future, the mechanism of action involved in these antinociceptive and anti-neuropathic effects should be explored.

### 2.34. Tithonia tubaeformis (Jacq.) Cass

*Tithonia tubaeformis,* best known as “acahual”, “palocote”, “gigantón” or “andan”, is an annual herb native to Mexico which grows throughout the entire country [203]. This native plant belongs to the Asteraceae family, one of the most studied families, although its phytochemical composition and its therapeutic uses have been not studied. This plant is commonly characterized by yellow flowers and is used to feed cattle as it grows in the form of undergrowth in an invasive way [204]. The phytochemical screening of this plant displayed the presence of bioactive compounds like tannins, phenols, flavonoids, coumarins, steroids and alkaloids. An in vitro assay demonstrated for the first time the anti-inflammatory activity of a methanolic extract from *T. tubaeformis* on the inhibition of porcine pancreatic elastase [205]. Furthermore, its analgesic effect was demonstrated with the administration of an oral increased dose of a hydroalcoholic extract (100 and 200 mg/kg) in the acid acetic-induced writhing and tail immersion tests. Furthermore, the same doses of hydroalcoholic extract reduced tactile allodynia and thermal hyperalgesia in vincristine-induced neuropathy [206]. Mice treated with 2000 mg/kg of methanolic extract from *T. tubaeformis* did not show behavioral changes, cyanosis, or other signs. Its oral acute toxicological profile was well tolerated. These results open a new field to investigate the therapeutic uses of this plant.

## 3. Future Directions in Preclinical Assays for Mexican Medicinal Plants

In this review, we attempt to contextualize the advances in preclinical assays of Mexican traditional plants and their derivates employed in the treatment of inflammatory or neuropathic pain conditions. Approximately thirty-seven plants have been studied regarding their antinociceptives properties (Table 1).

Considering the literature reports, this leads us to the following question: What is the future of medicinal plants from Mexico, in the field of pain managment? To understand the problem with translational pain research in the field of Mexican plants and their derivates it is necessary to understand the multidisciplinary factors that historically have not allowed traditional medicine to cross from preclinical to clinical research [207]. Thus, it is important highlight the fact that most of the studies listed in our review lack description of the mechanisms of action underlying these plants’ antinociceptive effects, as well as lacking toxicological tests or assays related to these plants’ side effects (Table 1). Relatedly, less than half of the studies attributed the plants’ antinociceptive effects to bio-compounds present in the plant, which suggests that most of the studies of the plants used extracts developed using methanol, ethanol, chloroform or other chemicals. Some of these extracts are not candidates for research addressed toward clinical studies. Several studies were performed with commercial compounds and were not necessarily isolated from the medicinal plant studied. Thus the characterization of the phytochemical composition of these plants is urgent, along with the linking of the plants’ effects to these compounds. The development of a commercial product for human use is based on standardized extracts, but it is important to understand their composition in order to define a marker compound for quality control. On the other hand, preclinical assays of Mexican plants and their antinociceptive properties mostly evaluated these plants using the formalin test, carrageenan-induced paw edema, acid acetic-induced writhing and thermal nociception tests, whereas only six studies focused on neuropathy models (Table 1). We consider that these points must be resolved in order to advance the study of pain treatment using Mexican medicinal plants. Finally, in agreement with other authors, we suggest the adoption of new models to evaluate inflammatory and neuropathic pain conditions, to provide effective and predictive behavioral animal models for future clinical trials [208,209].

## 4. Conclusions

Our data review suggested that most of the preclinical studies on Mexican folk plants used to treat pain address inflammatory pain, whereas only a few studies have investigated experimental models of neuropathy. On the other hand, further efforts are required to clarify and understand the mechanisms of action through which traditional plants and their derivatives exert their antinociceptive properties, as well as the toxic or adverse effects associated with their consumption. Finally, the preclinical evidence supports the common use of medicinal plants to treat pain ailments in Mexican folklore.

## Figures and Tables

**Table 1:** Mexican medicinal plants and their antinociceptive effects. This table contains a summary of preclinical studies with medicinal plants, as well as the possible mechanisms of action that underlie their antinociceptive effects.

Plant	Type of Extract	Experimental Model	Species	Possible Mechanism of Action	Reference
*Salvia divinorum*	Salvinorin A (11.6, 13.9, 18.5, 20.8 and 23.1 nmol, i.t.)	Tail flick test	Mice	Activation of kappa-opioid receptors	[32]
*Salvia divinorum*	Salvinorin A (0.5, 1.0, 2.0 and 4.0 mg/kg, i.p.)	Tail flick test Hot plate test Acetic acid-induced writhing	Male Swiss mice	Activation of kappa-opioid receptors	[33]
*Salvia divinorum*	Acetonic extract (30, 100 and 200 mg/kg, i.p.)	Sciatic loose nerve ligature-induced mechanical and thermal hyperalgesia Carrageenan-induced edema	Male Wistar rats	Activation of kappa-opioid receptors	[34]
*Salvia divinorum*	*Ethyl acetate extract* (31.6, 100 and 316 mg/kg, i.p.) and *Mixure salvinorins* (30 mg/kg, i.p)	Acetic acid-induced writhing Formalin test	Male and female Swiss albino mice	Opioids and 5-HT_1A_	[35]
*Heliopsis longipes*	Ethanolic extract (10, 30, 100 and 300 mg/kg. i.p.)	Thermal hyperalgesia	Balb/c mice	Not studied	[39]
*Heliopsis longipes*	Ethanolic extract (10, 30, 100 and 300 mg/kg, p.o.)	Carrageenan-induced hyperalgesia (hot box test)	Male Balb/c mice	Synergistic actions with diclofenac	[40]
*Heliopsis longipes*	Ethanolic extract (3, 10, 30 and 100 mg/kg, p.o.)	Acetic acid-induced writhing Hot plate	Male CD1^+^ mice	Not studied	[41]
*Heliopsis longipes*	Affinin (1, 3, 10, 100, 300 and 600 µg/region), Longipinamide A andLlongipenamide B compounds (0.1, 1, 10, 30 and 100 µg/region)	Formalin-induced orofacial pain	Female Swiss Webster mice	TRPV1	[44]
*Heliopsis longipes*	Ethanolic extract (10 mg/kg, i.p.) Afinin (1 mg/kg, i.p.)	Acetic acid-induced writhing Hot plate test	Male albino mice	Not studied	[45]
*Heliopsis longipes*	Acetonic extract (1, 10, 17.78, 31.6, 56.23 mg/kg, i.p.) Affinin (10, 17.78, 31.62, 56.23, 74.98 mg/kg, i.p.)	Capsaicin-induced hyperalgesia Acetic acid-induced writhing	Male ICR mice	Activation of nitric oxide, K^+^ channels, opioid, GABAergic and serotonergic system	[43]
*Artemisia ludoviciana*	Essential oil (1, 10, 31.6, 100 and 316 mg/kg, i.p.)	Hot plate test Formalin-induced hyperalgesia	Male ICR mice	Activation of Opioid system	[55]
*Caulerpa mexicana*	Sulphated polysaccharides (5, 10 and 20 mg/kg i.v.) (5, 10 and 20 mg/kg, s.c.) *	Acetic acid-induced writhing (no effect) Formalin-induced hyperalgesia (no effect) Carrageenan, dextran, histamine and serotonin -induced paw edema*	Male and female Swiss mice Male Wistar rats	Histamine is the main target of paw edema inflammation	[56]
*Caulerpa mexicana*	Methanolic extract Ethyl acetate extract Hexanic Chloroform extract (100 mg/kg, p.o.)	Formalin-induced hyperalgesia Acetic acid-induced writhing Hot plate test Carrageenan-induced peritonitis	Female Swiss mice	Not studied	[58]
*Agastache mexicana*	Hexane extract Ethyl acetate extract methanolic extract (100 mg/kg, i.p.) Ursolic acid compound (1, 3, 10, 30 and 100 mg/kg, i.p)	Acid acetic-induced writhing Formalin-induced hyperalgesia Intracolonic stimulation with capsaicin	Male and female Swiss albino mice and Wistar rats	Possible participation of cGMP and 5-HT_1A_ receptors.	[68]
*Agastache mexicana*	Ursolic compound (1–100 mg/kg) Acacetin compound (1–100 mg/kg, i.p.) (1–300 mg/kg p.o.)	Acetic acid-induced writhing	Male and female Swiss albino mice	Not studied	[69]
*Agastache mexicana*	Hexane extract Ethyl acetate extract Methanolic extract (10, 30, 100, 300 and/or 562.3 mg/kg or 1000 mg/kg, i.p)	Acetic acid-induced writhing Formalin-induced hyperalgesia Hot box test PIFIR model	Female Swiss albino mice	Not studied	[30]
*Ligusticum porteri*	Organic extract Aqueous extract Essential oil (31.6, 100 and 316 mg/kg, p.o.) *Z-*ligustilide compound *Z*-3-butylidenephthalide compound Diligustilide compound (10, 31.6, 56.2 mg/kg, p.o.)	Acetic acid- induced writhing Hot plate test	Male ICR mice	Not studied	[78]
*Ligusticum porteri*	Methanolic-chloroform extract (150, 275 and 300 mg/kg, i.p.)	Writhing test	Male ICR mice	Not studied	[79]
*Clinopodium mexicanum*	Aqueous extract (1, 5, 10 and 100 mg/kg, i.p.)	Hot plate test	Male Swiss Webster mice	Not studied	[83]
*Clinopodium mexicanum*	2 (S)-neopincirin (1, 10, 20 and 40 mg/kg, i.p.)	Hot plate test	Male Swiss Webster mice	GABAergic system was involved in the anxiolytic effect exerted by 2(S)-neopincirin	[84]
*Tilia americana* var *mexicana*	*Aqueous extract* *Quercentin* (30 and 100 mg/kg, i.p.)	Formalin-induced hyperalgesia PIFIR model	Male Wistar rats	Activation of 5-HT_1A_ receptors	[92]
*Acourtia thurberi*	Decoction (31.6, 100, 316.2 µg/paw and 31.6, 100 and 316.2 mg/kg, p.o.) Perezone (3.2, 10 and 31.6 µg/paw, s.c.) Mixure of α-pipitzol, β-pipitzul (3.2, 10 and 31.6 µg/paw, s.c.) 8-β-D-glucopyranosyloxy-4-methoxy-5-methyl-coumarin (3.2, 10 and 31.6 µg/paw, s.c.)	Formalin-induced hyperalgesia in normal and diabetic mice	Male ICR mice	Not studied	[95]
*Cyrtopodium macrobulbon*	Organic extract Aqueous extract (31.6, 100 and 316 mg/kg, p.o.)	Hot plate test Writhing test	Male ICR mice	Not studied	[96]
*Ternstroemia sylvatica*	Chloroform and Ethanolic extract (250 and 500 mg/kg i.p.)	Croton oil- and TAP-induced ear edema Carrageenan-induced paw edema Acid acetic-induced writhing test Formalin-induced hyperalgesia	Male ICR mice	Not studied	[99]
C*onyza filaginoides*	Organic extract (31, 100 and 316 mg/kg, p.o.) (1, 10, 30, 56, 100 µg/paw, s.c.)	Acetic acid-induced writhing Hot plate test Formalin-induced hyperalgesia in normal and diabetic mice	Male ICR mice	GABAergic and opioid pathways	[103]
*Choisya ternata*	*Essential oil* *Ethanolic extract* (10, 30 and 100 mg/kg, p.o.) Methyl N- methylanthranilate compound Isopropyl *N*-methylanthranilatePropyl *N*-methylanthranilate compound (0.3, 1 and 3 mg/kg, p.o.)	Acetic acid-induced writhing test Hot plate test	Male Swiss mice	Not studied	[107]
*Choisya ternata*	Isopropyl (ISOAN) compound Methyl (MAN) compound Propyl N-methylanthranilate (PAN) compound (0.3, 1 and 3 mg/kg, p.o.)	Formalin-induced hyperalgesia Capsaicin and Glutamate-induced nociception test Tail flick test Hot plate test	Male and female Swiss mice	K^+^_ATP_ channels (ISOAN) Adrenergic, nitrergic and serotoninergic pathways (ISOAN and MAN)	[109]
*Choisya ternata*	Essential oil ternanthranin (3, 10 and 30 mg/kg, p.o.)	Formalin-induced hyperalgesia Carrageenan-induced paw edema	Male Webster mice	Reduction of nitric oxide, TNF-α and IL-1β	[110]
*Mimosa albida*	Aqueous extract (2.5, 25 and 50 mg/kg, i.p.)	Hot plate test Acetic acid-induced writhing	Male ICR mice	Not involved opioid receptors	[111]
*Heterotheca inuloides*	HI-2 fraction (butanol fraction) from the aqueous extract	Acetic acid-induced writhing test Carrageenan-induced paw edema Dextran-induced paw edema	Female Wistar rats and male Swiss CD-1 mice	Not studied	[113]
*Heterotheca* *inuloides*	7-hydroxy-3,4-dihydrocadalin compound (10, 100 and 1000 µg/paw, s.c.)	Formalin-induced hyperalgesia Mechanical hyperalgesia (Randall–Selitto) Carrageenan-induced paw edema	Female Wistar rats	Activation the 5-HT_1A,_ 5-HT_1B,_ 5-HT_1D_, but not opioid receptors	[115]
*Heterotheca* *inuloides*	7-hydroxy-3,4-dihydrocadalin (0.03, 0.3, 3 and 30 mg/kg, p.o.)	Formalin induced hyperalgesia in diabetic neuropathy *	Female Wistar rats	Activation of serotonin, but not opioid receptors. Antioxidant effect (malondialdehyde)	[116]
*Calea zacatechichi*	Dichloromethane extract (200 mg/kg, p.o.)	Intracolonic instillation of mustard oil test Acetic acid-induced writhing	Male C57BL/6N mice	Not studied	[125]
*Calea zacatechichi*	Aqueous extract (200 mg/kg, p.o.)	Hot plate test Acetic acid-induced writhing	Male albino Swiss mice	Not studied	[126]
*Geranium bellum*	Acetone-aqueous extract (200, 400 and 800 µg/paw, s.c.) (75, 150 and 300 mg/kg, p.o.) Geraniin Corilagin Quercetin Ellagic acid (5–25 mg/kg, p.o.) Geraniin Quercentin Ellagic acid and Corilagin derivates from AC-AE *Geranium bellum*	Formalin-induced hyperalgesia Acetic acid-induced Hot plate test	Male Wistar rats Female CD1 albino mice	Not studied	[127]
*Sphaeralcea* *angustifolia*	*Chloroform extract* (400 mg/kg, i.p.)	Carrageenan-induced paw edema	Male Sprague-Dawley rats	Not studied	[136]
*Acacia farnesiana*	*Ethanol extract* (400 mg/kg, i.p.)	Carrageenan-induced paw edema	Male Sprague-Dawley rats	Not studied	[136]
*Rubus* *coriifolius*	Chloroform:methanolic extract (1:1) (400 mg/kg, i.p.)	Carrageenan-induced paw edema	Male Sprague-Dawley rats	Not studied	[136]
*Oenothera* *rosea*	Methanolic extract	Carrageenan-induced paw edema	Male Sprague-Dawley rats	Not studied	[136]
*Oenothera* rosea	*Ethanolic and* *Ethyl acetate extract* (50, 100 and 200 mg/kg, p.o.)	Acetic acid-induced writhing Hot plate test	Female NIH Swiss mice	Not studied	[151]
*Chamaedora* *tepejilote*	Aqueous extract	Carrageenan-induced paw edema	Male Sprague-Dawley rats	Not studied	[136]
*Astianthus viminalis*	Methanolic extract	Carrageenan-induced paw edema	Male Sprague-Dawley rats	Not studied	[136]
*Brickellia* *veronicaefolia*	Methanolic-chloroform extract (150, 300 and 600 mg/kg, p.o.)	Writhing test	Male ICR mice	Not studied	[79]
*Brickellia* *paniculata*	Methanolic extract	Carrageenan-induced paw edema	Male Sprague-Dawley rats	Not studied	[136]
*Justicia* *spicigera,*	Methanolic extract	Carrageenan-induced paw edema	Male Sprague-Dawley rats	Not studied	[136]
*Justicia spicigera*	Ethanolic extract (50, 100 and 200 mg/kg, p.o.)	Formalin-induced hyperalgesia test Hot plate test Tail flick test Acetic acid-induced writhing t	Male Balb/C mice	Not studied	[171]
*Lantana* *hispida*	Methanolic extract	Carrageenan-induced paw edema	Male Sprague-Dawley rats	Not studied	[136]
*Pittocaulon* *bombycophole* *P. velatum* *P. praecox* *P. hintonii*	Dichloromethane extract (100 mg/kg, i.p.)	Carrageenan-induced paw edema (no effect)	Male Wistar rats	Not studied	[181]
*Swietenia humilis*	Aqueous extract (10, 31.6, 56.2, 100 and 177 μ/paw, s.c.) Mexicanolide compound (0.5, 1, 2, 3 and 3.5 μg/paw, s.c.)	Formalin-induced hyperalgesia in diabetic mice	Male ICR mice	GABA_A_, 5-HT_2A/C_ and opiod receptors, as well as the nitrergic system.	[195]
*Ageratina pichinchensis*	3,5-diprenyl-4-hydroxyacetophenone compound (10, 32, 56 and 100 mg/kg, p.o.) (100, 128, 320 and 562 mg/kg, p.o.)	Carrageenan-induced thermal hyperalgesia Allodynia induced by spinal nerve ligation (L5/L6)	Male Wistar rats	Not studied	[198]
*Tithonia tubaeformis*	*Hydromethanolic extract* (100 and 200 mg/kg, p.o.)	Tail immersion test Acid acetic-induced writhing Vincristine-induced neuropathy	Balb/c mice	Not studied	[206]

## Data Availability

Not applicable.
